# Healthcare expenditures among community-dwelling adults with thyroid cancer in the United States: A propensity score matched analysis

**DOI:** 10.1016/j.heliyon.2019.e01995

**Published:** 2019-06-24

**Authors:** Sandipan Bhattacharjee, Moteb Khobrani, Ziyad Alrabiah, Jawad Bilal, Irbaz Bin Riaz

**Affiliations:** a Department of Pharmacy Practice and Science, College of Pharmacy, The University of Arizona, Tucson, AZ, USA; b College of Pharmacy, King Saud University, Riyadh, Saudi Arabia; c Health Outcomes & PharmacoEconomic Research (HOPE) Center, College of Pharmacy, The University of Arizona, Tucson, AZ, USA; d Department of Internal Medicine, The University of Arizona, Tucson, AZ, USA; e Clinical Pharmacy Department, College of Pharmacy, King Khalid University, Abha, Saudi Arabia

**Keywords:** Cancer research, Economics, Oncology, Thyroid cancer, Adults, Community-dwelling, Healthcare expenditures, Propensity score

## Abstract

**Objective:**

This study assessed the excess healthcare expenditures and factors associated with it among community-dwelling adults with thyroid cancer compared to non-cancer controls in the United States.

**Method:**

A retrospective, cross-sectional, matched case-control study design was used by pooling multiple years of Medical Expenditure Panel Survey (MEPS) data (2002–2012). The eligible study sample comprised of adults (age ≥18 years), who were alive during the calendar year and reported positive healthcare expenditure. The case group consisted of adults with thyroid cancer only while the control group consisted of adults who did not have any form of cancer. Total and subtypes of mean annual healthcare expenditures comprised the main study outcome. We also calculated the total and subtypes of out-of-pocket (OOP) expenditures as well as OOP as a percentage of household income. Ordinary Least Square (OLS) regressions on log-transformed expenditures were conducted to elucidate the influence of different factors on healthcare expenditures among adults with thyroid cancer.

**Results:**

The yearly average total healthcare expenditures among adults with thyroid cancer was significantly higher compared to propensity score matched controls ($9,585 vs. $5,830, p < 0.001). Similar observations were found in terms of inpatient, and outpatient expenditures. Functional status as well as comorbid conditions were significantly associated with excess expenditures. The yearly average total OOP expenditure for adults with thyroid cancer was significantly higher compared to matched controls ($1,425 vs. $974, p < 0.001), with major differences observed in inpatient OOP ($178 vs. $24, p = 0.003), outpatient OOP ($435vs. $256, p < 0.001), and prescription OOP ($554 vs. $423, p < 0.001) expenditures. There was a significant (p < 0.001) difference between the average OOP as a percentage of household income between adults with thyroid cancer (Mean: 7.54%, S.E: 1.52%) and matched controls (Mean: 5.80%, S.E: 0.47%).

**Conclusions:**

Our findings suggest that holistic care approach could be helpful to significantly reduce the economic burden in this population. Viable strategies such as limits on OOP costs are required to minimize this high OOP burden among cancer survivors and their families.

## 1 Introduction

Thyroid cancer is considered a fairly common type of cancer, with an estimated 637,115 individuals having the disease in the United States (US) in 2013 [1]. In 2016, the number of new thyroid cancer cases was estimated to be 64,300 [1], and the overall incidence of thyroid cancer was observed to increase 3% annually from 1974-2013 [2]. However, the 5-year survival for individuals with thyroid cancer was estimated to be 98.1% (2006–2012) [1]. Moreover, recent data showed that thyroid cancer has continuously increased in the last three decades all over the world, which can be attributed to sharp rise of use of more sensitive diagnostic procedures or a possible true increase due to the increased population exposure to some recognized or unrecognized carcinogens [3]. In addition, a recent report has projected the incidence of thyroid cancer in 2030 to be the fourth most common cancer based on the demographic changes, the average annual percentage changes in incidence and death rates [4].

A few recent studies have examined the burden of thyroid cancer in ex-US settings [5, 6, 7, 8, 9]. However, despite the increase in incidence of thyroid cancer, data on clinical and economic impact of thyroid cancer care is lacking in the US. Only a handful of studies in US examined the costs associated with thyroid cancer [10, 11, 12].It is crucial to estimate the burden of this disease for an appropriate utilization of specific treatment modalities and health care services in light of rising trends in incidence. Using National Cancer Institute's (NCI) Surveillance, Epidemiology, and End Results (SEER) 13 databases between 1992 -2009, a thyroid cancer cost analysis study was published in 2013 [10]. This study showed that the estimated lifetime cost for a hypothetical cohort of individuals with thyroid cancer is $34,723 per patient, and ranged from $58,660 to $33,463 for those with and without metastasis respectively [10]. In addition, the total cost for an incident cohort of thyroid cancer diagnosed in 2010 was approximately $1.4 billion and this number is projected to increase to more than $2.38 billion for the 2019 cohort. The total medical cost including diagnosis, treatment and management for the cohorts diagnosed between 2010 and 2019 is approximately $18.59 billion dollars. A recent study by Iadeluca et al. (2017) using different US data sources including Medical Expenditure Panel Survey (MEPS) 2011 data estimated the annual direct spending for thyroid cancer to be $5.4 billion (2011 US dollars) [11]. Another study using the SEER data conducted a stacked cohort cost analysis from 1985-2013 to estimate current and future healthcare expenditures attributable to well-differentiated thyroid cancer (WDTC) [12]. This study conducted by Lubitz et al. (2014) estimated the current societal costs of WDTC to be $1.6 billion in 2013 and predicted the future costs to be $3.5 billion in 2030 based on present incidence trends [12].

The existing studies on thyroid cancer expenditures does not provide a holistic view of the different factors associated with the excess expenditures. For example, the Aschebrook-Kilfoy et al. (2013) [10] study did not compare the healthcare expenditures of individuals with thyroid cancer with other controls. The Lubitz et al. (2014) [12] study focused specifically on the WDTC while the Iadeluca et al. (2017) [11] study only provided an annual estimate of the thyroid cancer expenditure. None of these studies examined the effect of co-occurring chronic conditions, health and functional status on healthcare expenditures among individuals with thyroid cancer. Assessing the impact of these factors on healthcare expenditures is important as existing literature has demonstrated the significant influence of these factors on healthcare use and expenditures [13, 14, 15, 16, 17, 18, 19]. Thus, the primary objectives of this study were to assess the excess healthcare expenditures (total and subtypes) and factors associated with it among a nationally representative sample of community-dwelling adults with thyroid cancer compared to non-cancer controls in the United States (US). Additionally we are presenting the total and subtypes of out-of-pocket (OOP) expenditures as well as OOP as a percentage of household income, which has not been reported any of the existing studies and will be helpful to understand the thyroid cancer burden on individuals as well their families.

## 2 Materials and methods

The data used in this study was publically available. Data can be downloaded from: https://meps.ahrq.gov/mepsweb/data_stats/download_data_files.jsp.

### 2.1 Study design and study sample

We adopted a retrospective, cross-sectional, matched case-control study design by pooling MEPS data from 2002 through 2012. We were not able to use MEPS data post 2012 despite being available due to change in coding system that did not allow for proper identification of adults with thyroid cancer. The study sample comprised of (i) adults aged 18 years or above; (ii) alive during the calendar year; and (iii) reported positive healthcare expenditure. We identified adults with thyroid cancer with Clinical Classification System (CCS) code of “36” (Cancer of thyroid). Crosswalk of CCS and International Classification of Diseases, Ninth Revision, Clinical Modification (ICD-9-CM) codes is published online by the Agency for Healthcare Research and Quality (AHRQ) [20]. The case group consisted of adults with only thyroid cancer (no other form of cancer). The control group consisted of adults who did not have any form of cancer and met all the study inclusion criteria. As the case and control group in this study are inherently different, we matched these two groups based on propensity score to minimize the chances of selection bias and make them comparable. Propensity score was generated based on age, gender, race/ethnicity, smoking status and Body Mass Index (BMI). These characteristics were chosen to match the two groups, as existing literature suggests that these characteristics are independent predictors of thyroid cancer [21, 22, 23, 24, 25]. Each case was matched to three controls (1:3) on propensity score using 8 to 1 greedy matching algorithm [26]. In the greedy matching algorithm, the case group (adults with thyroid cancer) is randomly selected at first followed by selecting the control subject (adults without any form of cancer) whose propensity score is closest to that of this randomly selected case. A similar iterative approach is used until all case group subjects are matched to control group subjects or until there are no other case group subjects left for whom a matched control group subject can be located. The reason for calling this process a “greedy” algorithm is because the nearest control group subject is selected for matching to the given case group subject, despite the possibility of that control group subject to better serve as a match for a subsequent case group subject [27]. Optimal matching algorithm, in which matches are formed to minimize the total within-pair difference of the propensity score, is a possible alternative to greedy matching [27]. We selected the greedy matching algorithm for our study over the optimal matching algorithm as an existing study [28] comparing these two algorithms found that optimal matching did no better than greedy matching in producing balanced matched samples and we have successfully implemented the greedy matching algorithm for several of our existing studies [29, 30, 31, 32, 33]. Certain co-occurring chronic conditions (such as diabetes [34]) are independent risk factor for thyroid cancer, but was not included in matching as this study assessed the impact of these co-occurring chronic conditions on healthcare expenditures among individuals with thyroid cancer. To demonstrate the balance between the case and control group post matching, we estimated the standardized mean differences (SMD) of the covariates on which the matching was conducted. While there is not a general consensus about the threshold of SMD, but based on prior literature we considered SMD <0.1 to demonstrate good balance between the case and control group after matching [35, 36]. The University of Arizona Institutional Review Board designated this study as “Human Subjects Review Not Required”.

### 2.2 Data source

We used publicly available MEPS (2002–2012) data for the purpose of this study [37]. MEPS data is collected by AHRQ and is a nationally representative survey of the US civilian noninstitutionalized population [38]. Sampling framework of the National Health Interview Survey (NHIS) is used by MEPS and to achieve nationally representative estimates, MEPS oversamples minority groups and individuals with disabilities [39]. We used the household component and medical conditions files of MEPS for this study. The household component consists data on demographics characteristics, health status, healthcare expenditure, healthcare service use, health insurance, employment, and incomes. The medical conditions file consists of self-reported data of each participant's medical conditions, which were coded using either CCS or ICD-9-CM diagnosis codes. The household and medical conditions files were merged using a unique identifier (DUPERSID).

### 2.3 Dependent variables

Total healthcare expenditure constituted the primary dependent variable of this study. Total healthcare expenditure was calculated as a sum of inpatient, outpatient, emergency room visits, prescribed medication use, home healthcare, and other (dental, vision, and other medical equipment and services) expenditures. Expenditure subtypes (such as inpatient, outpatient) were also a part of the dependent variable. Healthcare expenditures in this study were reported from various sources such as direct payments from individuals, private insurance, Medicare, Medicaid, Workers' Compensation, and miscellaneous other sources [40]. We also calculated the total and subtypes of OOP expenditures as well as OOP as a percentage of household income. Expenditures in this study were expressed in terms of 2012 US dollars (constant dollars) and were adjusted using the medical component of the annual consumer price index (CPI) obtained from the Bureau of Labor Statistics [41]. We transformed the expenditure data logarithmically in order to meet normality assumption of OLS, as the expenditure data was skewed.

### 2.4 Independent variables

We used the Ronal M. Andersen's Behavioral Model (ABM) of Health Services Use as a conceptual framework for this study [42]. The ABM consists of predisposing, enabling, need, healthcare environment and personal health practices factors, which influence healthcare use and expenditures. The independent variables used in this study were: age, gender, race/ethnicity, marital status, education, region, health insurance status, poverty status, MEPS year, activities of daily living (ADL), instrumental activities of daily living (IADL), functional disability, activities disability, perceived physical health status, perceived mental health status, BMI and co-occurring conditions. The specific chronic co-occurring conditions were identified using ICD-9-CM and CCS. The conditions included anxiety (CCS: 651); arthritis (CCS: 201–204); emphysema (CCS: 127 or ICD-9-CM: 491,492, 493, 496); type II diabetes (CCS: 49, 50); eye problems (CCS: 86, 88); gastroesophageal reflux disease or GERD (CCS: 138); heart diseases such as coronary heart disease, angina, myocardial infarction (CCS: 96, 97, 100–108); hypertension (CCS: 98, 99); depression (CCS: 69 or ICD-9-CM: 296, 311); osteoporosis (CCS: 206); stroke (ICD-9-CM: 430–438) and thyroid disorders (CCS: 48) [20].

### 2.5 Statistical analyses

We used t-tests to compare the mean healthcare expenditures between adults with thyroid cancer compared to matched controls. We compared the distribution of the predisposing, enabling, need, personal health practices and environmental factors before and after propensity score matching between adults with thyroid cancer and matched controls using chi-square test. We checked for the assumptions of Ordinal Least Square (OLS) regression such as independence of observations, homoscedasticity, linearity of parameters, and multi-collinearity for the logarithmically transformed expenditures. All these assumptions were met by the logarithmically transformed expenditure variables. An a-priori alpha of 0.05 was considered as the level of significance in all analyses. We conducted a series of OLS regressions on logarithmically transformed expenditures to elucidate the influence of different factors on healthcare expenditures among adults with thyroid cancer. In the first OLS regression model (Model 1), we adjusted for thyroid cancer, marital status, education, region, health insurance status, poverty status, and MEPS year. In the second OLS regression model (Model 2), we adjusted for ADL, IADL, functional disability, activities disability, perceived physical and mental health status in addition to the factors in Model 1. We adjusted for co-occurring chronic conditions in addition to the factors in Model 2 for the third OLS regression model (Model 3). We used semi-logarithmic equation (e^β^ – 1) to calculate the percent difference in costs between adults with thyroid cancer and matched controls [43]. We adjusted for the complex survey design of MEPS in all analyses to obtain nationally representative estimates [38]. We conducted all analyses using survey procedures in SAS version 9.4 software (SAS Institute Inc., Cary, NC, USA).

## 3 Results

### 3.1 Study sample

**Fig. 1:**
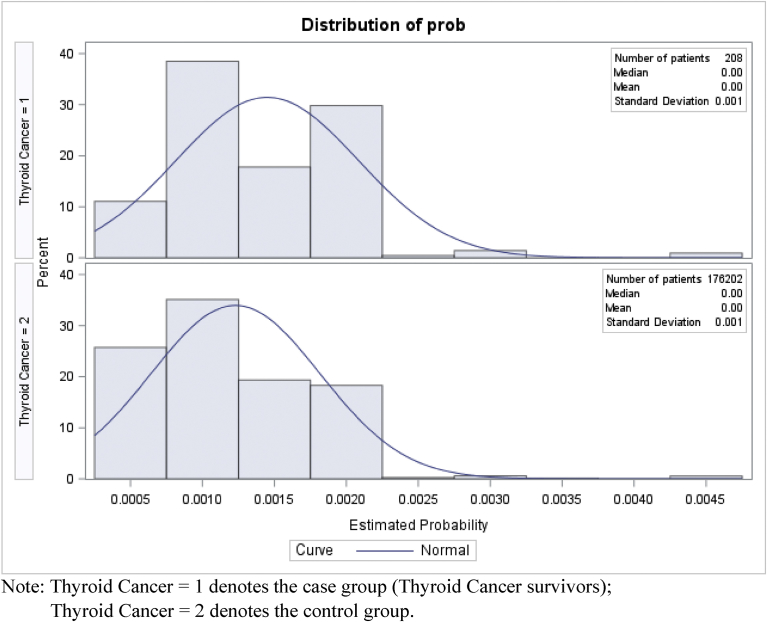
Propensity score distribution before matching. Note: Thyroid Cancer = 1 denotes the case group (Thyroid Cancer survivors); Thyroid Cancer = 2 denotes the control group.

**Fig. 2:**
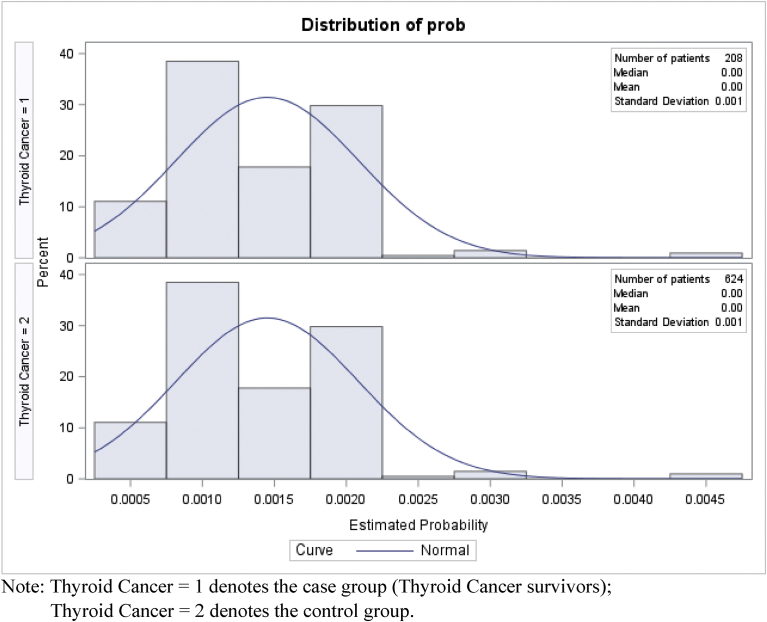
Propensity score distribution after matching. Note: Thyroid Cancer = 1 denotes the case group (Thyroid Cancer survivors); Thyroid Cancer = 2 denotes the control group.

Using MEPS 2002–2012 data, we had 208 adults with thyroid cancer and 176,202 adults without any form of cancer, who met all the study inclusion criteria before propensity score matching. Due to the high sample size of the non-cancer control group, we matched 208 adults with thyroid cancer to 624 adults without cancer based on propensity score, without losing sample from the case group. Table 1 presents the differences in individual level characteristics before and after propensity score matching. Prior to propensity score matching, the two groups were significantly different in terms of gender, poverty status, perceived physical and mental health status, IADL limitations, co-occurring chronic conditions and smoking status. Before matching, the proportion of females was higher in the thyroid cancer (70.9% vs. 55.8%) group compared to non-cancer control group. A greater proportion of adults with thyroid cancer reported higher income (52.1% vs. 42.6%), and fair or poor physical (18.8% vs. 13.6%) health status compared to non-cancer controls prior to matching. In terms of co-occurring chronic conditions, adults with thyroid cancer reported a higher proportion of eye disorders (8.3% vs. 3.7%), gastroesophageal reflux disorder (GERD) (16.7% vs. 7.4%), hypertension (42.4% vs. 26.1%), and thyroid disorders (26.1% vs. 7.4%) compared to non-cancer controls. A greater proportion of adult non-cancer controls reported being current smoker (19.0% vs. 11.6%) compared to adults with thyroid cancer. After propensity score matching, there were no statistically significant difference between adults with thyroid cancer and propensity score matched non-cancer controls in terms of gender and smoking status. However, significant differences existed after matching between the two groups in terms of poverty status, perceived physical and mental health status and some co-occurring chronic conditions. Figs. 1 and 2 exhibits the distribution of propensity score before and after matching respectively. Fig. 2 demonstrates the balance between the two groups after propensity score matching. The SMDs of gender, race/ethnicity, smoking status, and BMI were zero and that of the age group was 0.01, signifying that the case and control groups were well balanced in terms of the matching covariates (see Table 2).

**Table 1:** Characteristics of study sample before and after Propensity Score matching MEPS 2002–2012.

	Before matching			After matching		
	Thyroid cancer	No cancer		Thyroid cancer	No cancer	
	Wt. %	Wt. %	Sig	Wt. %	Wt. %	Sig
**Predisposing factors**						
**Age group**						
18–64	84.8	83.1		84.8	82.3	
65,+	15.2	16.9		15.2	17.7	
**Gender**						
Female	70.9	55.8	**	70.9	70.7	
Men	29.1	44.2		29.1	29.3	
**Race/Ethnicity**						
White	79.6	71.7		79.6	78.5	
Other	20.4	28.3		20.4	21.5	
**Enabling factors**						
**Marital status**						
Married	62.1	56.1		62.1	56.2	
Other	37.9	43.9		37.9	43.8	
**Education**						
LT HS	12.0	15.8		12.0	12.5	
HS	24.5	30.1		24.5	30.5	
> HS	63.5	54.1		63.5	56.9	
**Poverty status**						
Poor	5.1	10.6	*	5.1	8.0	*
Near Poor	16.9	16.4		16.9	16.8	
Middle Income	25.9	30.4		25.9	34.3	
High Income	52.1	42.6		52.1	40.9	
**Employment status**						
Employed	72.8	68.3		72.8	66.6	
Not employed	27.2	31.7		27.2	33.4	
**Insurance**						
Private	78.9	73.8		78.9	73.3	
Public	10.8	16.0		10.8	17.0	
Uninsured	10.4	10.2		10.4	9.6	
**Need factors**						
**Perceived Physical Health status**						
Ex/vgood	41.6	58.0	***	41.6	57.5	***
Good	39.6	28.3		39.6	26.4	
Fair/poor	18.8	13.6		18.8	16.1	
**Perceived Mental Health status**						
Ex/vgood	59.3	67.1	*	59.3	61.7	*
Good	35.1	25.1		35.1	27.9	
Fair/poor	5.7	7.8		5.7	10.4	
**ADL Limitations**						
Yes	1.9	2.7		1.9	3.2	
No	98.1	97.3		98.1	96.8	
(Contd.)						
**IADL Limitations**						
Yes	2.6	5.4		2.6	6.9	**
No	97.4	94.6		97.4	93.1	
**Activities disability**						
Yes	11.9	13.3		11.9	14.7	
No	88.1	86.7		88.1	85.3	
**Functional disability**						
Yes	29.1	26.2		29.1	27.0	
No	70.9	73.8		70.9	73.0	
**Chronic conditions**						
Anxiety	4.4	9.9	*	4.4	11.6	***
Arthritis	25.3	20.9		25.3	19.2	
Asthma	5.1	5.9		5.1	6.6	
Emphysema	9.2	10.5		9.2	9.1	
Diabetes	14.8	9.8		14.8	10.2	
Eye problems	8.3	3.7	*	8.3	4.2	
GERD	16.7	7.4	***	16.7	8.0	**
Heart disease	15.4	10.9		15.4	10.9	
Hypertension	42.4	26.1	***	42.4	25.6	***
Depression	14.6	11.3		14.6	14.4	
Osteoporosis	2.0	2.0		2.0	1.2	
Stroke	1.4	1.5		1.4	1.8	
Thyroid disorders	26.1	7.4	***	26.1	9.8	***
**Personal Health Practices**						
**BMI status**†						
Under or Normal	34.7	35.9		34.7	36.3	
Overweight	29.0	33.9		29.0	33.6	
Obese	31.6	28.4		31.6	26.8	
**Smoking status**						
Current smoker	11.6	19.0	*	11.6	13.8	
Other	88.4	81.0		88.4	86.2	
**External Environmental characteristics**						
**Metropolitan status**						
Metro	82.2	83.0		82.2	84.4	
Rural	17.8	17.0		17.8	15.6	
**Region**						
Northeast	24.2	18.7		24.2	16.6	
Mid-west	16.6	23.3		16.6	25.1	
South	40.5	35.7		40.5	35.3	
West	18.8	22.3		18.8	23.0	

Note: Based on 208 thyroid cancer survivors and 176,202 adults without cancer before matching; and 208 thyroid cancer survivors and 624 adults without cancer after matching among adults aged 18 years or older. The two groups were matched on age, gender, race/ethnicity, smoking status and body mass index.

Asterisks represent statistical significance between the two groups based on chi-square tests.

Abbreviations: MEPS: Medical Expenditure Panel Survey; Wt%: Weighted percentage; Sig: significant difference; Ex/vgood: excellent or very good; LT HS: less than high school; HS: high school; ADL: Activity of Daily Living; IADL: Instrumental Activity of Daily; GERD: Gastroesophageal Reflux Disorder.

***p < 0.001; ** 0.001 ≤ *p* < 0.01; *0.01 ≤ *p* < 0.05.

† Numbers do not add up to total numbers due to missing data.

**Table 2:** Standardized Mean Differences (SMDs) between case and control group after matching.

Variables	Standardized Mean Differences (SMDs)
Age group	0.01
Gender	0.00
Race/Ethnicity	0.00
Smoking status	0.00
Body Mass Index status	0.00

### 3.2 Expenditures

Table 3 provides the mean total and sub-types of expenditures (inpatient, ER, outpatient, prescription drugs, home healthcare, and other) along with standard errors among adults with thyroid cancer compared to non-cancer controls after propensity score matching. The yearly average total healthcare expenditures among adults with thyroid cancer was significantly higher compared to propensity score matched controls ($9,585 vs. $5,830, p < 0.001). Similar observations were found in terms of inpatient ($3,126 vs. $1,460, p < 0.05), and outpatient ($4,133 vs. $1,825, p < 0.001) expenditures. Even though we did not observe significant differences in terms of prescription expenditures in sub-types of expenditure, however, prescription expenditure was significant in the OLS models. The yearly average total OOP expenditure for adults with thyroid cancer was significantly higher compared to matched controls ($1,425 vs. $974, p < 0.001), with major differences observed in inpatient OOP ($178 vs. $24, p = 0.003), outpatient OOP ($435vs. $256, p < 0.001), and prescription OOP ($554 vs. $423, p < 0.001) expenditures (data not shown in tabular form). There was a significant (p < 0.001) difference between the average OOP as a percentage of household income between adults with thyroid cancer (Mean: 7.54%, S.E: 1.52%) and matched controls (Mean: 5.80%, S.E: 0.47%) (data not shown in tabular form).

**Table 3:** Mean total and types of expenditures (2012 US dollars) and standard errors of adults with Thyroid cancer compared to non-cancer controls after propensity score matching MEPS 2002–2012.

	Thyroid cancer (N = 208)		Non-cancer (N = 624)		
	Mean	S.E.	Mean	S.E.	Sig
Total	9,585	1,087	5,830	597	***
Inpatient	3,126	678	1,460	344	*
ER	179	64	157	22	
Outpatient	4,133	795	1,825	135	***
Prescription	1,587	139	1,319	91	
Other	488	72	459	46	

Note: Based on 208 thyroid cancer survivors and 624 adults without cancer after matching among adults aged 18 years or older. The two groups were matched on age, gender, race/ethnicity, smoking status and body mass index.

Asterisks represent statistical significance between the two groups based on t-tests.

Abbreviations: S.E.: Standard Error; Sig: Significant; ER: Emergency Room; HHA: Home Health Agency.

***p < 0.001; ** 0.001 ≤ *p* < 0.01; *0.01 ≤ *p* < 0.05.

Expressed in 2012 US Dollars.

### 3.3 Influence of different characteristics on expenditures

Table 4 demonstrates findings from the three different OLS regression models. In Model 1, when we adjusted for thyroid cancer, marital status, education, region, health insurance status, poverty status, and MEPS year, we observed that adults with thyroid cancer had 184%, 133%, 453% and 575% higher expenditures compared to propensity score matched non-cancer controls in terms of total, inpatient, outpatient and prescription expenditures respectively. In Model 2, when we adjusted for ADL, IADL, functional disability, activities disability, perceived physical and mental health status in addition to the characteristics in Model 1, we observed consistent findings, however, the incremental percentage differences decreased across different expenditure types. In the final model (Model 3), when we adjusted for co-occurring chronic conditions over and above the characteristics in Model 2, we observed similar findings but the incremental expenditures reduced further. For example, incremental expenditures in Model 3 compared to Model 2 were as follows: total expenditures (88% vs. 129%), inpatient expenditures (78% vs. 97%), outpatient expenditures (230% vs. 323%), and prescription expenditures (180% vs. 348%). The full Model 3 is presented in Table 5.

**Table 4:** Intercepts and parameter estimates for thyroid cancer from separate OLS regressions on logged healthcare expenditures (2012 US dollars) MEPS 2002–2012.

	Model 1				Model 2				Model 3			
	Beta	S.E.	Sig	% Diff	Beta	S.E.	Sig	% Diff	Beta	S.E.	Sig	% Diff
**Total**												
Intercept	6.350	0.033	***		5.984	0.032	***		7.723	0.052	***	
Thyroid cancer	1.043	0.124	***	184	0.827	0.112	***	129	0.633	0.119	***	88
No cancer	0.000	0.000			0.000	0.000			0.000	0.000		
**Inpatient**												
Intercept	0.705	0.043	***		0.329	0.046	***		2.541	0.153	***	
Thyroid cancer	0.846	0.251	***	133	0.679	0.238	**	97	0.575	0.237	*	78
No cancer	0.000	0.000			0.000	0.000			0.000	0.000		
**Outpatient**												
Intercept	3.685	0.056	***		3.286	0.056	***		5.276	0.083	***	
Thyroid cancer	1.710	0.181	***	453	1.442	0.166	***	323	1.195	0.172	***	230
No cancer	0.000	0.000			0.000	0.000			0.000	0.000		
**Prescription**												
Intercept	3.505	0.068	***		3.094	0.065	***		5.897	0.088	***	
Thyroid cancer	1.910	0.126	***	575	1.500	0.117	***	348	1.028	0.153	***	180
No cancer	0.000	0.000			0.000	0.000			0.000	0.000		

Note: Based on 208 thyroid cancer survivors and 624 adults without cancer after matching among adults aged 18 or older. The two groups were matched on age, gender, race/ethnicity, smoking status and body mass index. Emergency room and Home Health Agency expenditures are not presented as they were not statistically significantly different between these two groups.

Asterisks represent statistical significance between thyroid cancer and matched non-cancer controls based on t-tests.

Model 1 included thyroid cancer, marital status, education, region, health insurance status, poverty status, and MEPS year as independent variables.

Model 2 included thyroid cancer, marital status, education, region, health insurance status, poverty status, MEPS year, ADL, IADL, functional disability, activities disability, perceived physical health status, and perceived mental health status as independent variables.

Model 3 included thyroid cancer, marital status, education, region, health insurance status, poverty status, MEPS year, ADL, IADL, functional disability, activities disability, perceived physical health status, perceived mental health status and co-occurring conditions as independent variables.

S.E.: Standard Error.

***p < 0.001; ** 0.001 ≤ *p* < 0.01; *0.01 ≤ *p* < 0.05.

Sig: significant.

% Diff: Percent difference between thyroid cancer and matched non-cancer controls groups. The percent difference in expenditures between the two groups were calculated using semi-logarithmic equation (e^β^ – 1).

Abbreviations: OLS: Ordinary Least Squares.

**Table 5:** Intercepts and parameter estimates for Thyroid Cancer from Ordinary Least Square Regressions on logged healthcare expenditures (2012 US dollars) for the full model (Model 3) Medical Expenditure Panel Survey (2002–2012).

	Total Expenditures			Inpatient Expenditures			Outpatient Expenditures			Prescription Expenditures		
	Beta	S.E.	Sig	Beta	S.E.	Sig	Beta	S.E.	Sig	Beta	S.E.	Sig
Intercept	7.723	0.052	***	2.541	0.153	***	5.276	0.083	***	5.897	0.088	***
**Thyroid cancer**												
Yes	0.633	0.119	***	0.575	0.237	*	1.195	0.172	***	1.028	0.153	***
No	0.000	0.000		0.000	0.000		0.000	0.000		0.000	0.000	
**ADL limitations**												
Yes	0.354	0.029	***	0.816	0.093	***	−0.256	0.046	***	−0.042	0.047	
No	0.000	0.000		0.000	0.000		0.000	0.000		0.000	0.000	
**IADL limitations**												
Yes	0.174	0.023	***	0.410	0.063	***	−0.143	0.037	***	0.145	0.037	***
No	0.000	0.000		0.000	0.000		0.000	0.000		0.000	0.000	
**Activities disability**												
Yes	0.389	0.017	***	0.476	0.039	***	0.410	0.026	***	0.348	0.028	***
No	0.000	0.000		0.000	0.000		0.000	0.000		0.000	0.000	
**Functional disability**												
Yes	0.282	0.011	***	0.050	0.021	*	0.445	0.020	***	0.394	0.022	***
No	0.000	0.000		0.000	0.000		0.000	0.000		0.000	0.000	
**Perceived physical health status**												
Excellent/Very Good	−0.216	0.011	***	−0.202	0.021	***	−0.278	0.018	***	−0.442	0.019	***
Fair/Poor	0.196	0.014	***	0.468	0.035	***	0.184	0.021	***	0.105	0.022	***
Good	0.000	0.000		0.000	0.000		0.000	0.000		0.000	0.000	
**Perceived mental health status**												
Excellent/Very Good	0.040	0.011	***	0.076	0.023	***	0.056	0.019	**	0.001	0.019	
Fair/Poor	−0.022	0.017		−0.098	0.042	*	−0.064	0.028	*	−0.060	0.029	*
Good	0.000	0.000		0.000	0.000		0.000	0.000		0.000	0.000	
**Marital status**												
Married	0.137	0.009	***	0.151	0.016	***	0.261	0.017	***	0.208	0.017	***
Others	0.000	0.000		0.000	0.000		0.000	0.000		0.000	0.000	
**Education**												
> HS	0.281	0.012	***	0.061	0.023	**	0.459	0.023	***	0.263	0.022	***
HS	0.129	0.013	***	0.042	0.025		0.184	0.023	***	0.146	0.023	***
< HS	0.000	0.000		0.000	0.000		0.000	0.000		0.000	0.000	
**Metropolitan status**												
Metropolitan	0.013	0.014		0.009	0.024		−0.011	0.028		−0.152	0.031	***
Rural	0.000	0.000		0.000	0.000		0.000	0.000		0.000	0.000	
**Region**												
Mid-west	0.110	0.015	***	0.111	0.023	***	0.111	0.028	***	0.288	0.030	***
Northeast	0.084	0.018	***	0.025	0.025		0.151	0.031	***	0.218	0.036	***
South	0.010	0.014		0.098	0.022	***	−0.079	0.029	**	0.402	0.030	***
West	0.000	0.000		0.000	0.000		0.000	0.000		0.000	0.000	
**Income**												
High Income	0.219	0.016	***	−0.386	0.029	***	0.362	0.027	***	0.262	0.029	***
Middle Income	0.046	0.015	**	−0.303	0.028	***	0.168	0.026	***	0.055	0.025	*
Near Poor	−0.003	0.015		−0.167	0.028	***	0.054	0.025	*	0.016	0.025	
Poor	0.000	0.000		0.000	0.000		0.000	0.000		0.000	0.000	
**Insurance**												
Private	0.811	0.015	***	0.365	0.023	***	1.306	0.028	***	0.808	0.028	***
Public	0.784	0.018	***	0.568	0.031	***	1.249	0.032	***	0.918	0.032	***
Uninsured	0.000	0.000		0.000	0.000		0.000	0.000		0.000	0.000	
**Anemia**												
Yes	0.486	0.031	***	1.273	0.097	***	0.752	0.040	***	0.275	0.050	***
No	0.000	0.000		0.000	0.000		0.000	0.000		0.000	0.000	
**Anxiety**												
Yes	0.320	0.014	***	0.015	0.029		0.495	0.023	***	0.823	0.027	***
No	0.000	0.000		0.000	0.000		0.000	0.000		0.000	0.000	
**Asthma**												
Yes	0.142	0.023	***	0.008	0.055		−0.134	0.035	***	0.513	0.042	***
No	0.000	0.000		0.000	0.000		0.000	0.000		0.000	0.000	
**Arthritis**												
Yes	0.322	0.010	***	−0.023	0.023		0.662	0.017	***	0.475	0.019	***
No	0.000	0.000		0.000	0.000		0.000	0.000		0.000	0.000	
**Emphysema**												
Yes	0.227	0.017	***	0.085	0.040	*	0.470	0.027	***	0.741	0.028	***
No	0.000	0.000		0.000	0.000		0.000	0.000		0.000	0.000	
**Diabetes**												
Yes	0.443	0.013	***	0.144	0.035	***	0.301	0.021	***	1.198	0.021	***
No	0.000	0.000		0.000	0.000		0.000	0.000		0.000	0.000	
**Eye problems**												
Yes	0.507	0.017	***	0.039	0.049		1.041	0.026	***	0.694	0.029	***
No	0.000	0.000		0.000	0.000		0.000	0.000		0.000	0.000	
**GERD**												
Yes	0.537	0.014	***	0.178	0.037	***	0.520	0.024	***	1.227	0.024	***
No	0.000	0.000		0.000	0.000		0.000	0.000		0.000	0.000	
**Heart disease**												
Yes	−0.613	0.012	***	−1.010	0.037	***	−0.656	0.019	***	−0.704	0.021	***
No	0.000	0.000		0.000	0.000		0.000	0.000		0.000	0.000	
**Hypertension**												
Yes	0.449	0.010	***	0.049	0.022	*	0.498	0.016	***	1.705	0.016	***
No	0.000	0.000		0.000	0.000		0.000	0.000		0.000	0.000	
**Depression**												
Yes	0.432	0.013	***	0.120	0.029	***	0.557	0.022	***	1.114	0.024	***
No	0.000	0.000		0.000	0.000		0.000	0.000		0.000	0.000	
**Osteoporosis**												
Yes	−0.486	0.022	***	0.040	0.066		−0.712	0.035	***	−1.133	0.041	***
No	0.000	0.000		0.000	0.000		0.000	0.000		0.000	0.000	
**Stroke**												
Yes	−0.353	0.031	***	−1.210	0.120	***	−0.091	0.045	*	−0.224	0.050	***
No	0.000	0.000		0.000	0.000		0.000	0.000		0.000	0.000	
**Thyroid disorders**												
Yes	−0.386	0.013	***	−0.034	0.033		−0.653	0.023	***	−1.055	0.021	***
No	0.000	0.000		0.000	0.000		0.000	0.000		0.000	0.000	
**MEPS year**	−0.032	0.002	***	−0.014	0.003	***	−0.020	0.003	***	−0.091	0.003	***

Note: Based on 208 thyroid cancer survivors and 624 adults without cancer after matching among adults aged 18 or older. The two groups were matched on age, gender, race/ethnicity, smoking status and body mass index. Emergency room and Home Health Agency expenditures are not presented as they were not statistically significantly different between these two groups.

Asterisks represent statistical significance between thyroid cancer and matched non-cancer controls based on t-tests.

Model 3 included thyroid cancer, marital status, education, region, health insurance status, poverty status, MEPS year, ADL, IADL, functional disability, activities disability, perceived physical health status, perceived mental health status and co-occurring conditions as independent variables.

S.E.: Standard Error.

***p < 0.001; ** 0.001 ≤ *p* < 0.01; *0.01 ≤ *p* < 0.05.

Sig: significant.

Abbreviations: ADL: Activities of Daily Limitations; IADL: Instrumental Activities of Daily Limitations; OLS: Ordinary Least Squares; GERD: Gastroesophageal Reflux Disease; MEPS: Medical Expenditure Panel Survey.

## 4 Discussion

This study presents economic burden among community-dwelling adult thyroid cancer survivors in the US. Findings from this propensity score matched analysis demonstrated significantly higher economic burden among adults with thyroid cancer compared to matched controls in terms of average total, inpatient, outpatient, and prescription drug expenditures. Multivariate analyses revealed that several factors including gender, education, region, health insurance status, poverty status, ADL, functional disability, and co morbidities have significant influence on healthcare expenditures among adults with thyroid cancer.

There is a dearth of literature available on economic burden of thyroid cancer. Only handful of studies relevant to thyroid cancer expenditures are available in the US [10, 11, 12]. In our study, we reported the actual average yearly cost per patient with thyroid cancer residing in US communities. Furthermore, we also evaluated the impact of several factors including socioeconomic condition, mental and physical health and co-occurring chronic conditions on healthcare expenditures. Moreover, we are presenting the total and subtypes of out-of-pocket (OOP) expenditures as well as OOP as a percentage of household income.

Findings from this study suggest that outpatient expenditure among adults with thyroid cancer was one of the major contributors (accounting for nearly 43%) towards total healthcare expenditures. The high outpatient costs are explained by excellent prognosis of thyroid cancer. The 10-year disease-specific mortality that is associated with differentiated thyroid carcinoma is less than 5%. It is well established that initial stage of the cancer predicts overall survival in thyroid cancer patients. Fortunately, stage 1 and 2 represent 75% of all patients with thyroid disease. This high number is easily explained by inspection of American Joint Committee on Cancer/TNM Classification of Malignant Tumours (AJCC/TNM) classification that allocates all patient less than 45 years to stage 1 or 2 regardless of extent of disease [44]. Such a high survival rate mandates close surveillance of large population of thyroid cancer survivors. After curative thyroidectomy and radio-iodine ablation (RAI) treatment, these patients require Thyroid Stimulating Hormone (TSH) suppression therapy and measurement of serial thyroglobulin levels and TSH levels. Often, expensive imaging modalities such as neck Ultrasonography and MRI, Chest CT, RAI whole body scan and fluorodeoxyglucose (FDG)-positron emission tomography-(PET) are also necessary.

The observed inpatient expenditures were also close to outpatient expenditures (accounting for nearly 33%) which may reflect the cost of primary treatment modality (i.e. surgery) and administration of RAI for treating cancer and management of complications from treatment including surgery, radiation and chemotherapeutic medications requiring hospitalization. However, it should be emphasized that chemotherapy is rarely needed for treatment of differentiated thyroid cancers except for RAI-refractory disease.

Our study findings suggest that the total OOP as well as inpatient, outpatient and prescription OOP were higher among adults with thyroid cancer compared to matched non-cancer controls. While this is a unique addition from our study findings in the realms of thyroid cancer expenditures but this finding is intuitive. A recent report by the American Cancer Society Cancer Action Network (ACS CAN), estimated that the OOP for cancer treatment is approximately $4 billion (2014 data) [45]. Several factors that may be associated with high OOP burden among individuals with cancer include unforeseen and unmanageable expenditures such as requiring a treatment not covered by their plan, high co-insurance and deductibles, as well as possibility of seeking out-of-network care [45]. A study examining the associations between OOP costs and reduced and/or delayed treatment initiation observed that higher OOP costs were associated with higher rates of oral prescription abandonment and delayed initiation across cancers [46]. While the ACS CAN report [45] and the study by Doshi et al. (2018) [46] provide data from a general cancer perspective, but these issues are applicable to the higher OOP burden among thyroid cancer survivors and their families. In the light of the OOP burden discussion, it can be implied that financially viable strategies such as limits on OOP costs are required to minimize this high OOP burden among cancer survivors and their families.

Findings of OLS regression models suggest that after adjusting for health and functional status and co-occurring chronic conditions, the excess expenditure percentage of total healthcare reduced significantly. Existing studies suggest that fair or poor physical and mental health status [18] as well as functional limitations [19] lead to a high healthcare use and expenditures. Moreover, existence of a liner correlation between cancer and mental health status decline has been observed [14] that can lead to poor quality of life, medication non-compliance and increase risk of mortality [15]. All these negative outcomes generally lead to increased expenditure, which supports the finding of this study that mental and physical health status and co-occurring chronic conditions significantly influence healthcare expenditures among individuals with thyroid cancer. It implies that effective therapeutic approach should emphasize on optimization of co-occurring chronic conditions as well as physical and mental health for individuals with thyroid cancer. This is particularly true in the setting of thyroid cancer with high survival rate. The cancer-specific survival of patients with papillary thyroid cancer confined to the thyroid gland was 97% at 20 years even without treatment [47]. Although majority of these patients will not die from thyroid cancer, they live with the psychological burden of a cancer diagnosis.

These findings suggest that multidisciplinary approach is needed for close surveillance of individuals with thyroid cancer that includes the oncologist, primary care physician, pharmacist, clinical psychologist, physical and occupational therapists. It can significantly mitigate the economic burden in these patients. The Patient-Centered Medical Home (PCMH) is one of the noteworthy models of care that can serve thyroid cancer survivors well by promoting preventive services, providing holistic care and long-term surveillance post thyroid cancer treatment.

Some of the strengths of this study include the use of a robust study design such as the propensity score matching that helps to make the case (thyroid cancer) and control (non-cancer) comparable on observable charateristics. Moreover, this study provides estimates at national-level among community-dwelling adults with thyroid cancer and uses several key factors such as ADL, IADL, perceived physical and mental health status which have been shown to be independent predcitors of healthcare expenditure among cancer patients but is usually not available in other datasets. However, limitations of this study should be kept in mind while interpreting the findings. In this study, thyroid cancer was treated as a single entity, but histologically it is characterized into different types such as well differentiated papillary cancer with an excellent survival rate and anaplastic thyroid cancer with extremely poor prognosis. However, It should be recognized that most thyroid cancer is papillary thyroid carcinoma (accounts for approximately 85% of thyroid cancer cases) and therefore the fact that we have not accounted for different types is of lesser concern. This study did not have the following information in the MEPS database: stage of cancer detection, procedures performed, nature of care provider or specialty that can be used to examine difference as per hospital ratings or reputations, as well as information on cases of abandonment or relapse or death. Other limitations of this study include small sample sizeand not including recent treatment modalities such as new targeted therapies.

## 5 Conclusions

Given the rapid rate of increase in prevalence of thyroid cancer, it remains a significant clinical and economic burden on healthcare system. In addition to development of new diagnostic and therapeutic strategies, we should also focus on psychosocial impact of thyroid cancer that can significantly reduce the economic burden in this patient population. Moreover, it is also imperative that rigorous approach should be opted to treat the co-occurring chronic conditions. These measures mandate the organized efforts by a multidisciplinary team involved in the patient care. And finally, financially viable strategies such as limits on OOP costs are required to minimize this high OOP burden among cancer survivors and their families.

## Declarations

### Author contribution statement

Sandipan Bhattacharjee: Conceived and designed the experiments; Performed the experiments; Analyzed and interpreted the data; Contributed reagents, materials, analysis tools or data; Wrote the paper.

Moteb Khobrani, Ziyad Alrabiah, Jawad Bilal, Irbaz Bin Riaz: Conceived and designed the experiments; Performed the experiments; Analyzed and interpreted the data; Contributed reagents, materials, analysis tools or data; Wrote the paper.

### Funding statement

This research did not receive any specific grant from funding agencies in the public, commercial, or not-for-profit sectors.

### Competing interest statement

The authors declare no conflict of interest.

### Additional information

Data associated with this study has been deposited at https://meps.ahrq.gov/mepsweb/data_stats/download_data_files.jsp.
